# Rhinoplasty and YouTube: A Cross-Sectional Study to Assess the Quality, Dependability, and Reliability of Videos on Rhinoplasty

**DOI:** 10.7759/cureus.44804

**Published:** 2023-09-06

**Authors:** Nadia Djahanshahi, Keval B Patel, Helen A.O. Popoola-Samuel, Faris Fayyaz, Akash R

**Affiliations:** 1 Surgery, Saint George University, True Blue, GRD; 2 Surgery, Narendra Modi Medical College, Ahmedabad, IND; 3 Neurology, College of Health and Sciences, Rush University, Chicago, USA; 4 Surgery, Dow University of Health Sciences, Karachi, PAK; 5 Internal Medicine, Jagadguru Jayadeva Murugarajendra (JJM) Medical College, Davangere, IND

**Keywords:** rhinoplasty journey, rhinoplasty before and after, surgery, rhinoplasty, youtube

## Abstract

Introduction: Rhinoplasty, also referred to as a nose job or a reconstruction of the nose, is a surgical procedure that alters and reconstructs the nose for aesthetic or functional purposes. With the emergence of social media and modern internet accessibility, YouTube has gained popularity among users of all ages. Despite this, the accuracy and credibility of these videos and the information they include could be cause for concern.

Aims: This study intends to assess the competence, quality, and dependability of the information in the videos related to “Rhinoplasty surgery” on YouTube.

Methodology: This is a cross-sectional observational study that analyzes rhinoplasty information on YouTube. A Google Forms questionnaire was used to collect the data. The Global Quality Score (GQS), reliability score, and Video Power Index (VPI) were used to assess quality and reliability, and statistical analysis was performed using IBM Corp. Released 2012. IBM SPSS Statistics for Windows, Version 21.0. Armonk, NY: IBM Corp. The inclusion criteria were videos ranging from 1 minute to 20 minutes in duration, pertinent to the topic of rhinoplasty surgery, and in either English or Hindi.

Results: A total of 71 videos were analyzed, and 70 videos were included. A comparison of GQS, reliability score, and VPI based on the type of uploader was done by the Kruskal-Wallis test. The association between VPI, GQS, and reliability score with that of the uploader of the video was found to be statistically insignificant (p<0.05). Videos uploaded by healthcare organizations, news outlets, patients, or other relevant groups have the most GQS (4.5), whereas videos uploaded by hospitals have the lowest.

Conclusions: Compared to the type of uploader, videos have no statistically significant changes in quality, reliability, or video power, implying that the type of uploader does not necessarily impact the overall quality or reliability of the information presented in the investigated YouTube videos. While the majority of the videos addressed the indications of rhinoplasty and provided information about the etiology, there were gaps in discussing pre-procedural investigations and preventive measures. Due to our limitations, expanding the number of accounts used for search and increasing the number of videos might overcome the search algorithm.

## Introduction

Rhinoplasty, also referred to as a nose job or a reconstruction of the nose, is a surgical procedure that alters and reconstructs the nose for aesthetic or functional purposes [1]. When dissatisfied with their nose's size, shape, or functionality, many people elect to have this procedure. Additionally, it can assist in repairing trauma-related damage or genetic defects [2].

Before beginning the rhinoplasty process, the patient has a thorough consultation with a skilled plastic surgeon. Discussing the patient's worries and desired results during this meeting assists the surgeon in determining whether such objectives are realistically achievable [3]. The patient should start mentally and physically preparing before deciding whether to have surgery.

The surgeon will look at the shape of the patient's nose, skin quality, and general health to figure out the best way to help them. Once the evaluation is done, there are two main ways to do rhinoplasty surgery: open and closed [4]. During the surgery, the surgeon will reshape the bones, cartilage, and muscles to change the shape of the nose [5]. This could mean removing or adding tissue, changing the shape of the bridge or tip of the nose, straightening a crooked septum, or fixing other problems with how the nose works. The surgeon will use different techniques and tools to make the needed physical and functional changes. The patient must recover after surgical intervention for the most remarkable potential healing and results [6]. The time it takes for a patient to heal completely varies by case, although swelling and bruises usually decrease within several weeks. It is critical to strictly follow the post-operative care instructions to relieve the pain and suffering experienced during this period [7]. Plan periodic follow-up meetings with the surgeon to monitor the patient's progress and address any possible issues.

The primary aim of this study is to evaluate the information on the rhinoplasty procedure present on YouTube about rhinoplasty; and to evaluate the quality and reliability of these videos using the Global Quality Score (GQS) and reliability score.

## Materials and methods

A cross-sectional observational study was carried out in the month of June 2023 to determine the quality and reliability of uploaded information on rhinoplasty on YouTube. Each of the five authors was allotted a search term such as " Rhinoplasty", "Rhinoplasty surgery", "Rhinoplasty recovery", "Rhinoplasty before and after" and "Rhinoplasty journal". Each author evaluated 15 videos in the search term allotted to them. 

Videos in English or Hindi of a duration between 1 and 20 minutes and relevant to our topic of rhinoplasty were included in the study. The videos that did not meet one or more of the inclusion criteria were excluded from the study. Out of 75 videos evaluated, 70 videos were included in the study after being evaluated for inclusion-exclusion criteria and removing duplicates.

The videos were evaluated for baseline characteristics (likes, comments, duration since uploaded, and type of uploader), type of information related to rhinoplasty (pre-procedure, indications, before and after pictures, complications), and quality and reliability of information.

The Global Quality Score (GQS) and the Quality Criteria for Consumer Health Information (DISCERN) scales were used to evaluate the quality and reliability [8]. IBM Corp. Released 2012. IBM SPSS Statistics for Windows, Version 21.0. Armonk, NY: IBM Corp. was used for the statistical analysis of the 70 videos. Only the videos that satisfied the inclusion criteria were taken into consideration, and all other videos were excluded from the study.

The Global Quality Score (GQS) is a metric that is used to evaluate a video's overall quality and performance. It considers several variables, including video resolution, clarity, audio quality, and overall production value. Higher GQS ratings suggest that the video content is of higher quality and more engaging. The dependability score is a statistic that assesses video content's credibility and trustworthiness. The dependability score assists viewers in determining the authenticity of the video and making informed decisions about the information delivered. The Video Power Index (VPI) assesses a video's overall success and influence across many platforms. It considers things like viewership, engagement metrics (likes, shares, and comments), social media influence, and overall reach. VPI assists in determining a video's popularity, effectiveness, and possible viral impact. By assessing these scores, content creators can enhance the quality of their videos, advertisers can assess the efficacy of their campaigns, and consumers can make more informed decisions about the content they watch [9-11].

Statistical analysis was done using IBM Corp. Released 2012. IBM SPSS Statistics for Windows, Version 21.0. Armonk, NY: IBM Corp.

## Results

A total of 75 videos were evaluated, of which 70 met the inclusion criteria. Table 1 shows the characteristics of the analyzed videos. The analyzed 70 videos received a total of 48,996,635 views, with 359,103 likes, 17,214 dislikes, and 21,434 comments. Most of the videos, 58 (82.9%), were more than a year old. It was found that 31 (44.3%) of the videos were published by doctors, 23 (32.9%) by hospitals, and 16 (22.9%) by healthcare organizations, news outlets, patients, or other relevant groups.

**Table 1:** Characteristics of YouTube videos of rhinoplasty analyzed

Criteria	n (%)
Time since uploaded	
Less than one year (< 365 days)	12 (17.1%)
More than one year (> 365 days)	58 (82.9%)
Popularity	
Total no. of views	48996635
Total no. of likes	359103
Total no. of dislikes	17214
Total no. of comments	21434
Type of uploader	
Doctor	31 (44.3%)
Hospital	23 (32.9%)
Healthcare org./News/Patient/Others	16 (22.9%)

Table 2 gives an in-depth breakdown of the information disseminated in the analyzed videos. Fifty-one (72.86%) of the videos had information about pre- and post-procedural photographs; 48 (68.57%) videos discussed indications and post-procedural care; 46 (65.71%) videos described the anatomy of the involved area; and 28 (40%) videos described the prognosis after surgery.

**Table 2:** Type of information circulated about rhinoplasty on YouTube

Criteria	n (%)
Description of indications of rhinoplasty	48 (68.57%)
Information about the cause/etiology of nose problems?	45 (64.29%)
Information about investigations/tests prior to the procedure	20 (28.57%)
Information about prevention/vaccines	0 (0%)
Information about other treatment options	46 (65.71%)
Information about the mortality of surgery	2 (2.86%)
Information about rehabilitation after surgery	32 (45.71%)
Information about people/patient's sharing their own experience	24 (34.29%)
Information about parent sharing their experience with their family members	0 (0%)
The post has promotional content by a pharmaceutical company or by doctors?	20 (28.57%)
Description of anatomy of the involved area	46 (65.71%)
Description of pre-procedural care/preparation phase	36 (51.43%)
Description of post-procedural care	48 (68.57%)
Description of prognosis after surgery	28 (40%)
Description of pre- and post-procedural photographs	51 (72.86%)

Table 3 shows a comparison of GQS, reliability score, and VPI based on the type of uploader. Analysis to evaluate the association between VPI, GQS, and reliability scores with those of the type of uploader found scores to be statistically insignificant (p > 0.05). Videos uploaded by healthcare organizations, news outlets, patients, or other relevant groups have the highest GQS (4.5), whereas videos uploaded by hospitals have the lowest reliability score (3). This suggests that the type of uploader does not necessarily determine the overall quality or reliability of the information provided in the YouTube videos analyzed in our study.

**Table 3:** Comparison of GQS, reliability score, and VPI based on the type of uploader GQS: Global Quality Score, VPI: Video Power Index

	Doctors (n=31)	Hospital (n=23)	Healthcare org/News/Patient/Others (n=16)	P-value & test used
	Median (IQ1, IQ3)	Median (IQ1, IQ3)	Median (IQ1, IQ3)	Test Used: Kruskal-Wallis Test
VPI	46.86 (19.76, 187.43)	87.13 (17.5, 163.84)	107.9 (44.045, 196.42)	P-value = 0.275
GQS	4 (3, 5)	4 (3, 5)	4.5 (3, 5)	P-value = 0.798
Reliability Score	4 (3, 5)	3 (3, 4)	4 (3, 5)	P-value = 0.285

## Discussion

This study aimed to assess the characteristics of YouTube videos related to rhinoplasty and evaluate the information provided in these videos. These findings provided insights into the quality of information available on YouTube, which is a popular platform for seeking information on various topics, including medical procedures such as rhinoplasty.

In terms of the characteristics of the YouTube videos, it was found that the majority of the analyzed videos, 58 (82.9%), were uploaded more than one year ago, suggesting that the content available on YouTube for rhinoplasty may not be up to date. The findings of this present study are similar to those of the study conducted by Javidan et al., which reported a high proportion of outdated YouTube videos across different medical topics [12]. Outdated information can be misleading and potentially harmful to viewers seeking up-to-date information for decision-making.

The majority (44.3%) were uploaded by doctors, followed by hospitals (32.9%) and healthcare organizations, news outlets, patients, or other entities (22.9%). While videos uploaded by physicians may be expected to provide reliable and accurate information, the findings suggest that other sources, such as hospitals and healthcare organizations, also play a significant role in disseminating information about rhinoplasty on YouTube. This indicates that a diverse range of sources contribute to disseminating information about rhinoplasty on YouTube. This is consistent with the findings of a study by Strychowsky et al. that investigated YouTube videos on pediatric surgical procedures. They also identified physicians as the primary uploaders of such videos [13].

This study also discovered that most of the videos that discussed the indications of rhinoplasty were 48 videos (68.57%) and 45 videos (64.29%) on the cause or etiology of nose problems. This suggests that YouTube videos on rhinoplasty generally address the reasons behind the procedure and the problems it aims to rectify. However, there were notable gaps in the discussion of investigations or tests before procedure 20 (28.57%). This emphasizes the importance of providing comprehensive information, including diagnostic procedures and preventive measures, to ensure viewers are well-informed. This current study revealed that information on treatment options (46 [65.71%]) and rehabilitation after surgery (32 [45.71%]) was present in a significant proportion of the YouTube videos. This suggests that the videos provided viewers with alternative treatment options and guidance on postoperative care and recovery.

Researchers are assessing video quality and creator qualifications as a result of YouTube's increasing use as a key source of medical information [14,15]. This study provides a thorough analysis of YouTube videos as a source of information for rhinoplasty. It examines various aspects, such as the characteristics of the videos, the type of uploader, and the information covered in the videos, providing a comprehensive overview of the topic. The paper includes statistical results in the form of tables, allowing for a quantitative assessment of the data. This adds value to the study and enables comparisons with similar research conducted in the field. Comparisons with previous studies on YouTube videos related to medical procedures provide a broader context and facilitate a better understanding of the findings. This strengthens the validity and relevance of the research.

Limitations

This study had several limitations. The number of YouTube videos considered might be less than the total number of videos present on the platform. There can be interobserver variations that can affect the scoring in GQS as the tool evaluates videos based on six criteria, which may not capture all aspects of video quality. For example, the tool does not assess the credibility of the author, or the references used in the video. Moreover, the videos have limited opportunities for viewer engagement, which may affect their effectiveness in promoting rhinoplasty education.

## Conclusions

The study conducted a comprehensive analysis of YouTube videos related to rhinoplasty, focusing on their characteristics, the information provided, and the type of uploaders.

In conclusion, this study sheds light on the characteristics, quality, and authenticity of the content uploaded on YouTube related to rhinoplasty. It highlights the need for improved information coverage while emphasizing the role of diverse uploaders. The findings contribute to our understanding of the quality and reliability of such videos, providing valuable insights for both viewers and content creators. Future research could address the identified limitations and explore other aspects of YouTube videos related to medical procedures.
